# Comparative Outcomes of First-Line Chemotherapy for Metastatic Pancreatic Cancer Among the Regimens Used in Japan: A Systematic Review and Network Meta-analysis

**DOI:** 10.1001/jamanetworkopen.2021.45515

**Published:** 2022-01-31

**Authors:** Yuki Takumoto, Yuriko Sasahara, Hiroto Narimatsu, Manabu Akazawa

**Affiliations:** 1 Department of Public Health and Epidemiology, Meiji Pharmaceutical University, Tokyo, Japan; 2 Center for Outcomes Research and Economic Evaluation for Health, National Institute of Public Health, Saitama, Japan; 3 Department of Clinical Oncology, Yamagata Prefectural Central Hospital, Yamagata, Japan; 4 Department of Genetic Medicine, Kanagawa Cancer Center, Yokohama, Kanagawa, Japan; 5 Cancer Prevention and Cancer Control Division, Kanagawa Cancer Center Research Institute, Yokohama, Kanagawa, Japan; 6 Graduate School of Health Innovation, Kanagawa University of Human Services, Kawasaki, Kanagawa, Japan

## Abstract

**Importance:**

Various first-line chemotherapy treatment regimens for patients with metastatic pancreatic cancer have been approved in Japan, including gemcitabine (GEM); fluorouracil, leucovorin, irinotecan, and oxaliplatin combination (FOLFIRINOX); GEM plus albumin-bound paclitaxel (GEM+NPTX), and S-1 (tegafur + gimeracil + oteracil). However, direct comparisons of these chemotherapy regimens are limited.

**Objective:**

To assess the short-term and long-term outcomes associated with first-line chemotherapy regimens for metastatic pancreatic cancer compared with chemotherapy regimens recommended in Japanese guidelines.

**Data Sources:**

In this systematic review and network meta-analysis, the bibliographic databases PubMed, Cochrane Library, and Web of Science, as well as medical journals published between January 1, 2002, and December 31, 2018, were searched for clinical trials comparing chemotherapy regimens.

**Study Selection:**

Randomized 2-arm clinical trials evaluating first-line chemotherapy for advanced or metastatic pancreatic cancer were included.

**Data Extraction and Synthesis:**

The Preferred Reporting Items for Systematic Reviews and Meta-analyses (PRISMA) Extension Statement for Reporting of Systematic Reviews Incorporating Network Meta-analyses of Health Care Interventions was followed for data abstractions. Data were pooled using a random-effects model. The SIGN 50 Quality Assessment Instrument was used to assess the risk of bias and overall study quality of the selected trials.

**Main Outcomes and Measures:**

The primary end point was overall survival (OS), and the secondary end point was progression-free survival (PFS) compared with GEM for first-line chemotherapy for metastatic pancreatic cancer. The Kaplan-Meier curve of GEM from the literature and the estimated hazard ratios (HRs) were used to model the long-term associations to calculate the area under the curve (AUC) (person-months) for OS and PFS of each chemotherapy. Sensitivity analyses with multiple functional models were conducted to confirm the long-term estimations.

**Results:**

A total of 22 regimens (25 studies) for OS and a total of 18 regimens (21 studies) for PFS were identified from literature. The total number of participants was 10 186, with 5856 male (57.5%) and 4330 female (42.5%). The FOLFIRINOX and GEM+NPTX regimens were associated with reduction in the risk of death, with an HR of 0.57 (95% CI, 0.41-0.79) and 0.72 (95% CI, 0.55-0.95) compared with GEM, respectively. The curve estimation also showed that FOLFIRINOX had the largest AUC for survival at 15.49 person-months (range, 13.84-15.51 person-months), followed by GEM+NPTX with 12.36 person-months (range, 10.98-12.59 person-months), GEM+ERLO with 10.84 person-months (range, 9.66-11.23 person-months), S-1 with 8.44 person-months (range, 8.26-9.74 person-months), and GEM with 8.10 person-months (range, 7.93-9.38 person-months).

**Conclusions and Relevance:**

The results of this network meta-analysis support the relative short-term and long-term outcomes associated with first-line chemotherapy for metastatic pancreatic cancer used clinically in Japan.

## Key Points

**Question:**

What are the comparative short-term and long-term outcomes associated with first-line chemotherapy regimens for patients with metastatic pancreatic cancer, and how do they compare with chemotherapy regimens recommended in Japanese guidelines?

**Findings:**

In this systematic review and network meta-analysis of 25 randomized clinical trials assessing first-line chemotherapy regimens for advanced or metastatic pancreatic cancer, results were consistent with and complementary to the Japanese guidelines for pancreatic cancer treatment. Oxaliplatin, irinotecan, fluorouracil, and leucovorin combination (FOLFIRINOX) and gemcitabine plus albumin-bound paclitaxel (GEM+NPTX) had better outcomes associated with reduced risk of death as primary chemotherapy for pancreatic cancer than the unapproved drugs in Japan.

**Meaning:**

In this network meta-analysis, a similar trend in the curve estimation was found in randomized clinical trials, indicating that the values are consistent between the short and long terms; results suggest that FOLFIRINOX and GEM+NPTX can be regarded as regimens with the best outcomes compared with regimens not approved in Japan.

## Teaser

This systematic review and network meta-analysis compares the short-term and long-term outcomes associated with first-line chemotherapy regiments used in Japan for patients with metastatic pancreatic cancer.

## Introduction

Pancreatic cancer, a leading cause of cancer deaths in high-income countries, is one of the deadliest carcinomas worldwide.^1,2^ It has the fourth highest site-specific cancer mortality rate in Japan,^3,4,5^ and it is asymptomatic in the early stages and difficult to detect.^6^ Therefore, it is often diagnosed only in the advanced or metastatic stage when discomfort or symptoms arise in the affected area. The recommended treatment for metastatic pancreatic cancer is chemotherapy in Japan.^7^ Several chemotherapeutic regimens have been developed as treatment options for patients with advanced and metastatic pancreatic cancer since 1997, when gemcitabine (GEM) was reported to prolong survival in patients with pancreatic cancer.^8^ A chemotherapy regimen consisting of fluorouracil, leucovorin, irinotecan, and oxaliplatin (FOLFIRINOX), introduced in 2011, is reportedly a superior treatment for pancreatic cancer with a good performance status. It has shown significance in overall survival (OS) and progression-free survival (PFS) compared with GEM alone. However, FOLFIRINOX has a potential to cause some serious adverse events.^9^ The Japan Pancreas Society Clinical Practice Guidelines for Pancreatic Cancer 2019 described FOLFIRINOX as “a therapy that should be administered to patients up to 75 years old with a good performance status and organ function, such as bone marrow function.”^7^ Additionally, GEM plus albumin-bound paclitaxel (GEM+NPTX) statistically significantly improved the OS and PFS in patients with metastatic pancreatic cancer in 2013.^10^ Although the median OS and PFS durations in GEM+NPTX were slightly inferior to those of the FOLFIRINOX phase 3 trial, the results showed a good balance between comparative efficacy and safety in terms of mild hematologic toxic effects. For these reasons, GEM+NPTX is expected to be a new treatment for metastatic pancreatic cancer. Furthermore, tegafur + gimeracil + oteracil (S-1), an approved chemotherapy regimen for pancreatic cancer in Japan and Korea, is recommended by the Japan Pancreas Society Clinical Practice Guidelines for Pancreatic Cancer 2019.^7^ Although several clinical trials have shown that S-1 is comparable to GEM, it is better tolerated than the FOLFIRINOX and GEM+NPTX therapies owing to its minor adverse events.^11,12,13,14^ Moreover, since S-1 can be administered orally, it is thought to be beneficial as an alternative therapy in terms of reducing the treatment burden on patients.

The choice of chemotherapy regimen should be based on the patient's condition and treatment needs because of the diverse characteristics of the chemotherapy regimens currently available in Japan. However, the efficacy of recommended chemotherapies for pancreatic cancer has mainly been evaluated in clinical trials using only GEM as a control treatment, and the position of the chemotherapies recommended in Japan are unclear. Moreover, these approved chemotherapies in Japan have only been evaluated in limited phase 2 and phase 3 trials, with no study examining the long-term sustainability of the comparative outcomes associated with each chemotherapy. We compared short-term and long-term efficacies of first-line chemotherapies for metastatic pancreatic cancer.

## Methods

This systematic review and network meta-analysis was conducted in accordance with the Preferred Reporting Items for Systematic Reviews and Meta-analyses (PRISMA) reporting guideline Extension Statement for Reporting of Systematic Reviews Incorporating Network Meta-analyses of Health Care Interventions.^15^

### Literature Search and Search Procedure

A systematic review was conducted using PubMed, Cochrane Library, Web of Science, and medical journals for randomized clinical trials published between January 1, 2002, and December 31, 2018 (eTable 1 in the Supplement). In the screening procedure, 2 reviewers (Y.T. and Y.S.) independently searched abstracts and selected them according to the search criteria. The SIGN 50 Quality Assessment Instrument, a checklist for assessing the quality of randomized clinical trials developed by the Scottish Intercollegiate Guidelines Network, was used to assess the risk of bias and overall study quality of the selected trials.^16^

### Search Criteria

The inclusion criteria were randomized 2-arm clinical trials for patients with pancreatic cancer, first-line chemotherapy for advanced or metastatic pancreatic cancer, the number of patients in each arm greater than or equal to 50, and at least 1 of the following 2 outcomes: OS or PFS. The exclusion criteria were history of radiotherapy in the patient's treatment or having at least 50% of patients with nonmetastatic pancreatic cancer in each treatment arm.

### Data Extraction

The following information was extracted from search results and used as a criteria for determining whether comparability was possible. Bibliographic information was extracted for author name, year of publication, journal name, title, inclusion criteria, exclusion criteria, primary end point, and secondary end point. Background information was extracted for stratification factors, number of patients, treatment regimen, age, performance status, cancer stage, median duration of treatment, and the established dose used in the trials.

### Outcome Measures and Target Population

The primary end point was OS, and the secondary end point was PFS. We defined short-term outcomes as the range of observation periods in clinical trials and long-term outcomes as the range that can be estimated beyond the observed period of clinical trials. We also calculated the short-term outcomes associated with each regimen in a network meta-analysis (NMA) for each end point and estimated the long-term outcomes associated with each regimen using parametric functions.

Regarding the short-term assessment, we defined the median hazard ratio (HR) and 95% CIs of the OS and PFS of the comparator against the control as the end points. The control arm was GEM, and the comparator arm was all chemotherapy regimens used for metastatic pancreatic cancer except for GEM.

Regarding the long-term outcome assessment, we defined the area under the curve (AUC) of the estimated PFS and OS curves for each treatment regimen as the end points. The control drug was GEM, and the comparator drugs were 4 chemotherapy regimens recommended in the Japanese guidelines for the treatment of metastatic pancreatic cancer, namely, FOLFIRINOX, GEM+NPTX, S-1, and GEM+erlotinib (ERLO).

### Statistical Analysis

#### NMA Method

To compare the short-term outcomes of all chemotherapy regimens, including those without a direct comparison, we performed a bayesian NMA using WinBUGS 1.4 (MRC Biostatistics Unit).^17^ In the NMA, all treatment options with the same end points were compared simultaneously.^18,19,20,21,22^ Only multiple direct comparisons are performed in a traditional meta-analysis. However, if at least 1 arm in every clinical trial was linked to an arm in another clinical trial, these were integrated into the NMA. Moreover, the NMA was based on the assumption that all relative effect size indexes of the comparative medicines are homogeneous. We calculated the posterior probability distribution from the collected pooled HRs from the literature and the set prior probability distribution using the bayesian method. Using this posterior probability distribution, we evaluated the difference between drug regimens for each set evaluation index. The half-informative prior for between-trial SD (SD ~ *Log normal distribution[*0, 0.32^−2^]) was set as the prior distribution.^23^ It is suggested that setting an uninformative prior may lead to excessive expansion of 95% CIs and other measures in comparison with a small number of trials. We conducted the analysis using a random-effects model based on the NICE Single Technology Appraisal.^24^ We performed a 2-tailed test, with a *P* value of .05 considered as statistically significant.

#### Long-term Outcome Estimation Method

Using the methods described in the literature by Latimer^25^ and in a technical report by the Decision Support Unit of NICE,^26^ we estimated curves for long-term OS and PFS of 5 regimens being used in Japan. First, we selected trials with high representation from systematic review results to estimate OS and PFS curves for each regimen. This selection was performed by 2 oncology experts (Y.S. and H.N.). They selected 1 representative clinical trial per chemotherapy regimen based on the number of eligible patients, patient background, and study design (5 clinical trials in total). Second, we digitized the Kaplan-Meier (KM) curves of OS and PFS for the GEM arm in selected trials using WebPlotDigitizer by Rohatgi (version 4.3)^27^ to simulate individual patient data. Based on the study design, several patients, and patient characteristics of each trial, we confirmed the homogeneity of the GEM arm in each trial and integrated the digitization data of the GEM arms in the respective PFS and OS periods. The digitized data of the integrated GEM arms were fitted to 6 parametric functions (exponential, lognormal, log-logistic, Weibull, gamma, and Gompertz). We calculated the Akaike information criterion and bayesian information criterion of the estimated curves using 6 functional models to choose the best functional model. Moreover, we checked the visual fit of the KM curve. Specifically, we visually checked the fit of the 6 curves to the KM curve and selected a maximum of 3 curves. We then selected the curve with the lowest Akaike information criterion and bayesian information criterion from the selected curves as the best-fitting curve (base case). The effect size curve estimation for each chemotherapy regimen was analyzed by inserting the relative comparison results of OS-HR and PFS-HR calculated in the NMA into the base case. The estimated period of the curve began from the start of treatment to week 50 (referring to the OS of FOLFIRINOX, which was the longest OS from the follow-up period in selected trials of each chemotherapy). The AUC was calculated from the estimated PFS and OS curves to evaluate the comparison between chemotherapies. Analyses were performed using R software (version 4.0.4)^28^ with the flexsurv (version 2.0) and survHE (version 1.1.2) packages.^29,30^

#### Sensitivity Analysis

We estimated the PFS and OS curves using unselected parametric functions in the curve estimation for the integrated GEM group. We also calculated the AUC and checked the deviation from the base case.

## Results

### Description of Eligible Trials

**Figure 1.**
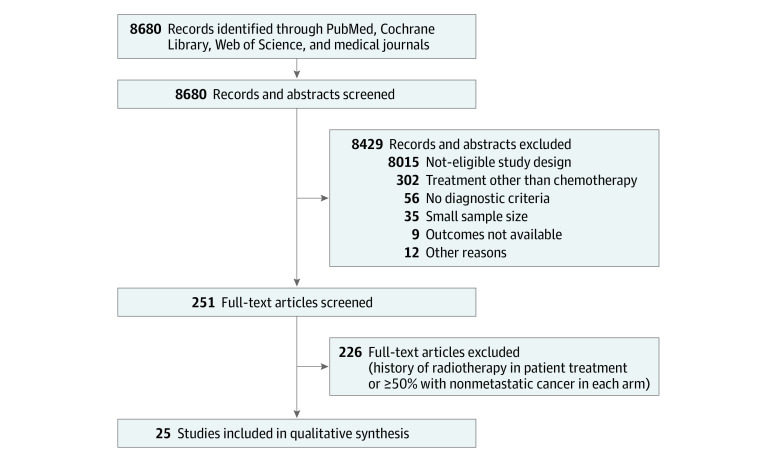
Preferred Reporting Items for Systematic Reviews and Meta-Analyses (PRISMA) Flow Diagram of Study Selection Process

A total of 251 of 8680 reports were screened by title and abstract. Twenty-five were selected by full-text screening; 226 were excluded. Twenty-five reports were finally included in the analysis (Figure 1).^9,10,11,13,14,31,32,33,34,35,36,37,38,39,40,41,42,43,44,45,46,47,48,49,50^ The total number of participants was 10 186, with 5856 male (57.5%) and 4330 female (42.5%). Most included trials were 2-arm comparisons with GEM (eFigure 1 in the Supplement). Nine of 25 articles (36.0%) were considered to be of high quality. Based on the results, 25 articles (22 chemotherapy regimens) were included in the analysis for OS, and 21 (18 chemotherapy regimens) were analyzed for PFS (eTable 2 in the Supplement).

### Results of the Outcomes in NMA

**Figure 2.**
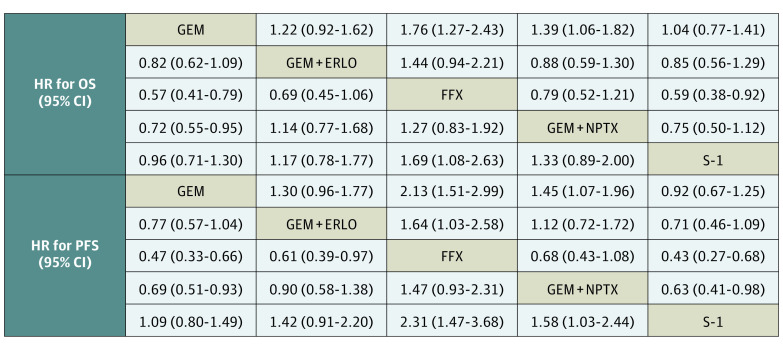
Pooled Estimates of the Network Meta-analysis of Recommended Chemotherapy Regimens in Japan ERLO indicates erlotinib; FFX, fluorouracil + leucovorin + irinotecan + oxaliplatin; GEM, gemcitabine; HR, hazard ratio; NPTX, albumin-bound paclitaxel; OS, overall survival; PFS, progression-free survival; S-1, tegafur + gimeracil + oteracil.

The relative HRs of OS for each chemotherapy regimen against the GEM arm suggested that the FOLFIRINOX (HR, 0.57; 95% CI, 0.41-0.79) and GEM+NPTX (HR, 0.72; 95% CI, 0.55-0.95) arms showed statistically significant reductions in the risk of death (Figure 2). The OS-HR of GEM against S-1 was 1.04 (95% CI, 0.77-1.41). Of the unapproved regimens in Japan, none surpassed GEM in reducing the risk of death (eTable 3A in the Supplement). The OS-HR rankings of each regimen among the 22 chemotherapy regimens used worldwide were as follows: the FOLFIRINOX arm ranked first; GEM+NPTX, third; GEM+ERLO, fifth; and S-1, eleventh; all ranked higher than the GEM arm (eFigure 2 in the Supplement). The relative HRs of PFS of each chemotherapy regimen against the GEM arm suggested that the FOLFIRINOX (HR, 0.47; 95% CI, 0.33-0.66) and GEM+NPTX (HR, 0.69; 95% CI, 0.51-0.93) arms showed statistically significant reductions in the progression risk (Figure 2). The PFS-HR of GEM against S-1 was 0.92 (95% CI, 0.67-1.25). Of the unapproved regimens in Japan, none surpassed GEM in reducing the risk of progression (eTable 3B in the Supplement). The PFS-HR rankings of each regimen among the 18 chemotherapy regimens used globally were as follows: the FOLFIRINOX arm ranked first; GEM+NPTX, fifth; and GEM+ERLO, sixth. Only the S-1 arm was ranked lower than the GEM arm and was ranked 18th (eFigure 2 in the Supplement). In addition, when comparing treatment regimens other than GEM used in Japan, no statistically significant difference in HR existed for both OS and PFS when comparing the OS of FOLFIRINOX and GEM+NPTX; however, this comparison suggested that the PFS-HR may tend to be lower with FOLFIRINOX (FOLFIRINOX vs GEM+NPTX: HR, 0.68; 95% CI, 0.43-1.08). Moreover, the PFS-HR of FOLFIRINOX and GEM+NPTX for S-1 was 0.43 (95% CI, 0.27-0.68) and 0.63 (95% CI, 0.41-0.98), respectively, and the OS-HR of FOLFIRINOX for S-1 was 0.59 (95% CI, 0.38-0.92) (Figure 2).

### Model Selection

Two oncology experts (Y.S. and H.N.) selected trials for analysis of each of the 4 chemotherapy regimens set as the control arm for model estimation (Table 1). Digitization and model estimation of KM curves for OS and PFS in the 4 trials were performed based on the Akaike information criterion, bayesian information criterion, and visual fit. Therefore, we rated the log-logistic function as the best fitting function for all chemotherapy regimens and outcomes (eTable 4 and eFigure 3 in the Supplement).

**Table 1.** Background of Patients in Major Clinical Trials of Recommended Chemotherapy Regimens in Japan

Variable	Patients, No. (%)							
	Moore et al,^31^ 2007		Conroy et al,^9^ 2011		Von Hoff et al,^10^ 2013		Ueno et al,^11^ 2013	
	GEM (n = 284)	GEM + ERLO (n = 285)	GEM (n = 171)	FFX (n = 171)	GEM (n = 430)	GEM + NPTX (n = 431)	GEM (n = 277)	S-1 (n = 280)
Age, median (range), y	64 (36-92)	64 (37-84)	61 (34-75)	61 (25-76)	62 (27-86)	63 (32-88)	NA	NA
Age range, y								
<66	NA	NA	NA	NA	NA	NA	134 (48)	145 (52)
≥66	NA	NA	NA	NA	NA	NA	143 (52)	135 (48)
Sex								
Male	57	48	61	62	60	57	61	61
Female	43	52	39	38	40	43	39	39
PS score								
ECOG = 0 (KPS = 100)	30	30	39	37	NA (16)	NA (16)	65	64
ECOG = 1 (KPS = 80 ~ 90)	52	51	61	62	NA (76)	NA (77)	35	36
ECOG = 2 (KPS = 70)	18	19	0	1	NA (8)	NA (8)	0	0
Pancreatic tumor location								
Local	25	24	0	0	0	0	24	24
Metastatic	75	76	100	100	100	100	76	76

Abbreviations: ECOG, Eastern Cooperative Oncology Group; ERLO, erlotinib; FFX, fluorouracil + leucovorin + irinotecan + oxaliplatin; GEM, gemcitabine; KPS, Karnofsky Performance Status; NA, not applicable; NPTX, albumin-bound paclitaxel; PS, performance status; S-1, tegafur + gimeracil + oteracil.

### Comparison With AUC of Other Estimations

**Figure 3.**
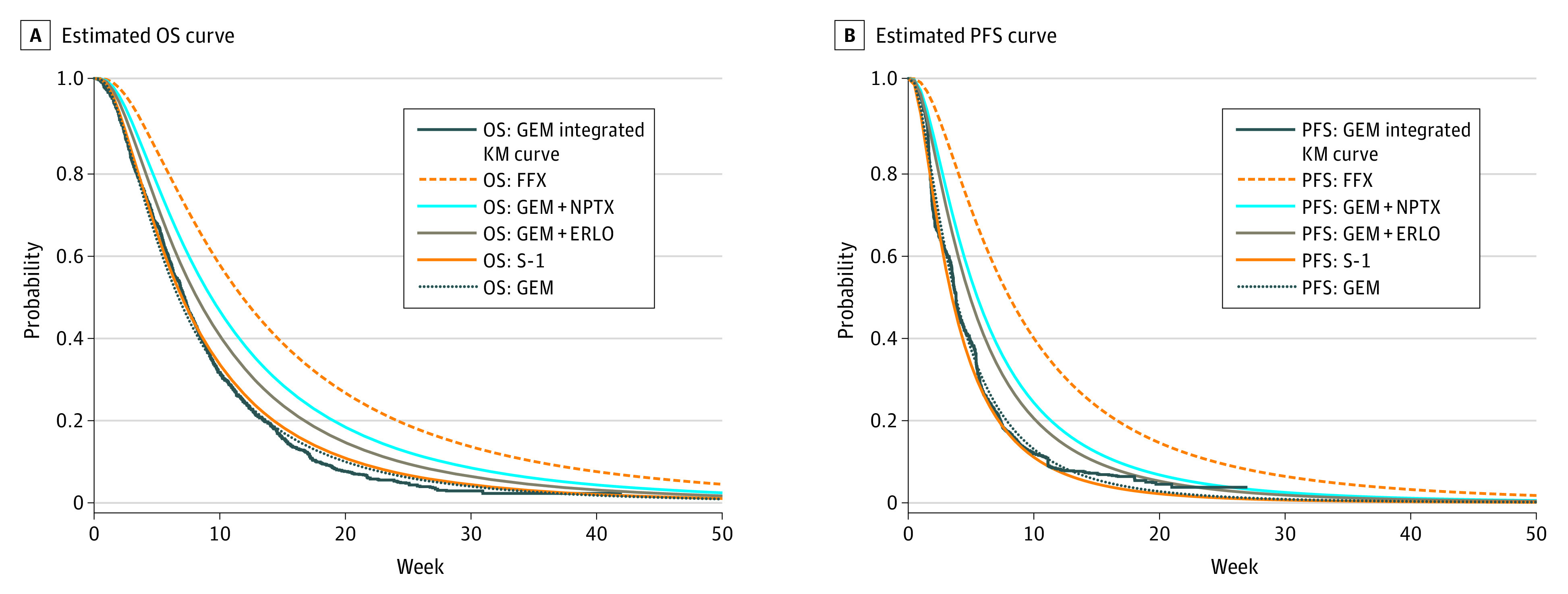
Comparison With Estimated Survival Curves ERLO indicates erlotinib; FFX, fluorouracil + leucovorin + irinotecan + oxaliplatin; GEM, gemcitabine; KM, Kaplan-Meier; NPTX, albumin-bound ‎paclitaxel; OS, overall survival; PFS, progression-free survival; S-1, tegafur + gimeracil + oteracil.

The AUC of each chemotherapy regimen performed using the selected functional model was highest in the OS curve for the FOLFIRINOX group at 15.49 person-months (range, 13.84-15.51 person-months), followed by the GEM+NPTX arm at 12.36 person-months (range, 10.98-12.59 person-months); GEM+ERLO arm at 10.84 person-months (range, 9.66-11.23 person-months), S-1, 8.44 person-months (range, 8.26-9.74 person-months); and GEM, 8.10 person-months (range, 7.93-9.38 person-months) (Table 2 and Figure 3). The PFS curves were similar, but the FOLFIRINOX arm had the highest PFS at 11.61 person-months (range, 11.52-12.46 person-months), followed by the NPTX arm at 8.16 person-months (range, 8.07-8.55 person-months); GEM+ERLO, 6.94 person-months (range, 6.88-7.42 person-months); GEM, 5.35 person-months (range, 5.29-5.72 person-months); and S-1, 5.19 person-months (range, 5.11-5.48 person-months).

**Table 2.** AUCs of Curve Estimation (Person-Months)

Regimen	OS		PFS	
	AUC, base case (range)	Rank	AUC, base case (range)	Rank
FFX	15.49 (13.84-15.51)	1	11.61 (11.52-12.46)	1
GEM+NPTX	12.36 (10.98-12.59)	2	8.16 (8.07-8.55)	2
GEM+ERLO	10.84 (9.66-11.23)	3	6.94 (6.88-7.42)	3
S-1	8.44 (8.26-9.74)	4	5.19 (5.11-5.48)	5
GEM^a^	8.10 (7.93-9.38)	5	5.35 (5.29-5.72)	4

Abbreviations: AUC, area under the curve; ERLO, erlotinib; FFX, fluorouracil + leucovorin + irinotecan + oxaliplatin; GEM, gemcitabine; NPTX, albumin-bound ‎paclitaxel; OS, overall survival; PFS, progression-free survival; S-1, tegafur + gimeracil + oteracil.

^a^ Integrated arm.

### Sensitivity Analysis

The long-term estimates of each regimen by other unselected parametric functions did not deviate substantially from the base case. The AUCs for PFS and OS were similar to those of the base case as shown in the AUC ranges in Table 2.

## Discussion

The results of this NMA may help in clarifying the position of chemotherapy regimens for pancreatic cancer used worldwide in terms of short-term outcomes. Based on NMA results, not only FOLFIRINOX and GEM+NPTX, which are approved in Japan, but also many unapproved regimens in Japan showed a statistically significant decrease in the relative HR for both OS and PFS compared with GEM. The 2021 National Comprehensive Cancer Network guidelines also suggest that FOLFIRINOX and GEM+NPTX are highly effective regimens and may be considered the preferred regimens for primary treatment of metastatic pancreatic cancer in patients with a good performance status, and the results of the evaluation of the NMA in this study may support this recommendation.^51^

We also compared the outcomes associated with each treatment regimen against S-1 from the treatment regimens recommended by the Japanese guidelines. The findings suggests that FOLFIRINOX and GEM+NPTX are significantly superior to S-1 in terms of OS and PFS. In contrast, there was no statistical significance in the finding of GEM compared with S-1 in OS-HR and PFS-HR. Moreover, the results of the collection, selection, and integrated analysis of the literature in the NMA showed results similar to those of Gresham et al.^52^ The NMA results were not biased, and were generally appropriate from the aspect of reproducibility.

In the curve estimation using the NMA results, we calculated the AUCs for PFS and OS for each chemotherapy regimen recommended in Japan and compared the AUCs for OS and PFS. We found that the estimated results were generally consistent with the NMA results. One of the reasons for the consistency between the NMA and the curve estimation results may be that the clinical trials collected in this study had a high rate of participants experiencing some adverse events during the study period, which may have been a factor in the curve estimation calculated using the KM curves of the clinical trials.

### Limitations

This study has limitations. First, it was difficult to verify that no heterogeneity between indirect and direct comparisons existed, which is an assumed hypothesis of NMAs. Most of the clinical trials about pancreatic cancer and chemotherapy regimens were analyzed using GEM as a control arm. We addressed this limitation by comparing the results with those of previous NMAs and clinical trials. In the future, it may be necessary to validate the NMA when clinical trials of networks without direct comparative trials are conducted. Second, potential bias from the racial background of participants in the clinical trials could have been present. The only chemotherapy regimen with a 2-arm randomized comparison trial unique to Japan was S-1. Therefore, racial differences, treatment status in each country, criteria and timing of exacerbations, and palliative care after treatment discontinuation may have been associated with the PFS and OS duration. In contrast, FOLFIRINOX, GEM+NPTX, and GEM+ERLO have been evaluated in a single-arm trial in Japan, with results consistent with those of global trials. In addition, the NMA calculated the relative HR using effect sizes in each clinical trial and did not reflect the absolute effect size in OS and PFS based on the patient background in each clinical trial. Third, the curve estimation in this study was performed by digitizing the KM curve from clinical trials to create pseudopatient-specific data; therefore, the analysis was not based on the exact duration and censoring of PFS and OS for each patient in the actual clinical trials. However, in this study, we integrated the digitized OS and PFS duration per patient from the KM curves in clinical trials and analyzed them after confirming that the variation of the estimated curve was not associated with the change in AUC. In addition, the clinical trials integrated into the curve estimation of this analysis were not entirely consistent, suggesting that differences in trial design and patient background for each trial may affect the long-term outcomes. In particular, only the clinical trial in the S-1 arm did not have patients with an Eastern Cooperative Oncology Group score of 2 or a Karnofsky Performance Status of 70 and only 76% of patients in both arms had metastatic pancreatic cancer. The S-1 trial was one of the trials used in this analysis that included patients with less severe disease, suggesting that the inclusion may have been a factor in the estimation of PFS and OS. Conversely, it is difficult to make a comprehensive comparison of chemotherapy regimens using only completely identical clinical trials in pancreatic cancer, for which evidence from clinical trials is limited. It is essential to evaluate the validity of the results by confirming the differences among clinical trials used for the analysis by selecting the analysis method according to the research purpose and confirming consistency by sensitivity analysis.

We were able to clarify the ranking of comparative outcomes associated with pancreatic cancer chemotherapy regimens used in Japan and other countries worldwide by comparing them with that of GEM. The comparative outcomes reported displayed a similar curve estimation, suggesting that the values may be consistent between the short and long terms. In particular, the results suggest that FOLFIRINOX and GEM+NPTX may be regarded as regimens associated with the best outcomes compared with regimens not approved in Japan. In addition, S-1 was associated with outcomes similar to GEM, although the AUC for OS and PFS was smaller compared with FOLFIRINOX and GEM+NPTX. We suggest that S-1 may be a viable option for patients who cannot receive FOLFIRINOX or GEM+NPTX or after receiving the second-line treatment. The possibility that biases among clinical trials in the NMA have not been permanently eliminated warrants further accumulation of evidence and additional analyses.

## Conclusions

In this systematic review and network meta-analysis, the outcomes associated with first-line chemotherapy regimens for metastatic pancreatic cancer approved in Japan and overseas were compared. FOLFIRINOX and GEM+NPTX were associated with the best outcomes compared with regimens not approved in Japan.

## Notes

Supplement. **eTable 1:** Search Strategy **eTable 2.** List of Selected Publications by Systematic Review **eTable 3.** Pooled Estimates of the Network Meta-analysis in Recommended Chemotherapy in the World **eTable 4.** AIC / BIC in Estimated GEM Curve **eFigure 1.** Network Diagram for Network Meta-analysis **eFigure 2.** HR Ranking of Each Regimen in NMA **eFigure 3.** Model Estimation of the KM Curves for OS and PFS
eReferences
