# Supplementary material for: Comparative Outcomes of First-Line Chemotherapy for Metastatic Pancreatic Cancer Among the Regimens Used in Japan: A Systematic Review and Network Meta-analysis
Source: JAMA Netw Open. 2022 Jan 31;5(1):e2145515. doi: 10.1001/jamanetworkopen.2021.45515 (PMC8804927; doi:10.1001/jamanetworkopen.2021.45515)
Supplement: Supplement. — eTable 1: Search Strategy eTable 2. List of Selected Publications by Systematic Review eTable 3. Pooled Estimates of the Network Meta-analysis in Recommended Chemotherapy in the World eTable 4. AIC / BIC in Estimated GEM Curve eFigure 1. Network Diagram for Network Meta-analysis eFigure 2. HR Ranking of Each Regimen in NMA eFigure 3. Model Estimation of the KM Curves for OS and PFS eReferences [file jamanetwopen-e2145515-s001.pdf]

Supplemental Online Content

Takumoto Y, Sasahara Y, Narimatsu H, Akazawa M. Comparative outcomes of first-line chemotherapy for metastatic pancreatic cancer among the regimens used in Japan: a systematic review and network meta-analysis. *JAMA Netw Open.* 2022;5(1):e2145515. doi:10.1001/jamanetworkopen.2021.45515

eTable 1. Search Strategy

eTable 2. List of Selected Publication by Systematic Review

eTable 3. Pooled Estimates of the Network Meta-analysis in Recommended Chemotherapy in the World

eTable 4. AIC / BIC in Estimated GEM Curve

eFigure 1. Network Diagram for Network Meta-analysis

eFigure 2. HR Ranking of Each Regimen in NMA

eFigure 3. Model Estimation of the KM Curves for OS and PFS

eReferences

This supplemental material has been provided by the authors to give readers additional information about their work.

© 2022 Takumoto Y et al. *JAMA Network Open*.

eTable 1: Search strategy

**Contents**	**Num**	**Search detail**
Pancreas cancer	#1	([advanced OR metastatic] AND pancreatic AND cancer) OR ([adenocarcinoma OR pancreas* OR malign*] AND neoplasm)
RCT	#2	“randomized controlled trial”
Language	#3	English and Japanese
Date Publication	#4	Between 1 January 2002 and 31 December 2018
Search strategy	#1 and #2 and #3 and #4	

© 2022 Takumoto Y et al. *JAMA Network Open*.

eTable 2: List of selected publication by systematic review

Aurhor	Year	Study design	Arm1	Arm2	Number of Patient in Arm 1 (ITT)	Number of Patient in Arm 2 (ITT)	Outcomes	Sign 50
Conroy T [1]	2011	RCT-open label	Gemcitabine	FOLFIRINOX	171	171	OS	+
							PFS	
Von Hoff DD [2]	2013	RCT - open label	Gemcitabine	Gemcitabine	430	431	OS	+
				Nab-paclitaxel			PFS	
Ueno H [3]	2013	RCT - open label (3 arms)	Gemcitabine	S-1 / Gemcitabine + S-1	277	280 / 275	OS	+
							PFS	
Nakai Y [4]	2012	RCT - open label	Gemcitabine	Gemcitabine	53	53	OS	+
				S-1			PFS	
Sudo K [5]	2014	RCT - open label	Gemcitabine	Gemcitabine	50	51	OS	+
				S-1			PFS	
Moore MJ [6]	2007	RCT - double blinded	Gemcitabine	Gemcitabine	284	285	OS	++
				Erlotinib			PFS	
Louvet C [7]	2005	RCT-open label	Gemcitabine	Gemcitabine	156	157	OS	+
				Oxaliplatin			PFS	
Kindler HL [8]	2010	RCT - double blinded	Gemcitabine	Gemcitabine	302	300	OS	++
				Bevacizumab			PFS	
Herrmann R [9]	2007	RCT - open label	Gemcitabine	Gemcitabine	159	160	OS	+
				Capecitabine			PFS	
Reni M [10]	2005	RCT - open label	Gemcitabine	PEFG	47	52	OS	+
							PFS	
Cunningham D [11]	2009	RCT - open label	Gemcitabine	Gemcitabine	266	267	OS	+
				Capecitabine			PFS	
Philip PA [12]	2010	RCT - open label	Gemcitabine	Gemcitabine	371	372	OS	++
				Cetuximab			PFS	
Van Cutsem E [13]	2009	RCT - double blinded	Gemcitabine	Gemcitabine	301	306	OS	++
			erlotinib	Erlotinib Bevacizumab			PFS	
	2004		Gemcitabine	Gemcitabine	347	341	OS	++

© 2022 Takumoto Y et al. *JAMA Network Open*.

Van Cutsem E [14]		RCT - double blinded		Tipifarnib			PFS	
Rougier P [15]	2013	RCT - double blinded	Gemcitabine	Gemcitabine	275	271	OS	+
				Aflibercept			PFS	
Colucci G [16]	2010	RCT - open label	Gemcitabine	Gemcitabine	199	201	OS	+
				Cisplatin			PFS	
Lee HS [17]	2017	RCT - open label	Gemcitabine	Gemcitabine	106	108	OS	+
				Capecitabine			PFS	
O'Neil BH [18]	2015	RCT - open label	Gemcitabine	Gemcitabine	54	106	OS	+
				Rigosertib			PFS	
Deplanque G [19]	2015	RCT - double blinded	Gemcitabine	Gemcitabine	175	173	OS	++
				Masitinib				
Yamaue H [20]	2015	RCT - double blinded	Gemcitabine	Gemcitabine	53	100	OS	++
				Elpamotide				
Ozaka M[21]	2012	RCT - open label	Gemcitabine	Gemcitabine	59	53	OS	+
				S-1				
Bramhall SR [22]	2002	RCT - double blinded	Gemcitabine	Gemcitabine	119	120	OS	+
				Marimastat			PFS	
Oettle H [23]	2005	RCT- open label	Gemcitabine	Gemcitabine	282	283	OS	+
				Pemetrexed				
Kindler HL [24]	2011	RCT - double blinded	Gemcitabine	Gemcitabine	316	314	OS	++
				Axitinib			PFS	
Gonçalves A [25]	2012	RCT - double blinded	Gemcitabine	Gemcitabine	52	52	OS	++
				Sorafenib			PFS	

© 2022 Takumoto Y et al. *JAMA Network Open*.

eTable 3: Pooled estimates of the network meta-analysis in recommended chemotherapy in the world.

A) Hazard rations for overall survival

Comparison arm	Control arm	OS-HR (95%CL)
FOLFIRINOX	Gemcitabine	0.57 [0.41 - 0.79]
S-1	Gemcitabine	0.96 [0.71 - 1.30]
Gemcitabine + S-1	Gemcitabine	0.81 [0.66 - 0.99]
Gemcitabine + nab-paclitaxel	Gemcitabine	0.72 [0.55 - 0.95]
Gemcitabine + erlotinib	Gemcitabine	0.82 [0.62 - 1.09]
Gemcitabine + bevacizumab + erlotinib	Gemcitabine	0.73 [0.49 - 1.09]
Gemcitabine + capecitabine	Gemcitabine	0.85 [0.71 - 1.01]
Gemcitabine + cisplatin	Gemcitabine	1.10 [0.81 - 1.49]
Gemcitabine + oxaliplatin	Gemcitabine	0.83 [0.60 - 1.14]
Gemcitabine + cetuximab	Gemcitabine	0.94 [0.72 - 1.24]
Gemcitabine + axitinib	Gemcitabine	1.01 [0.73 - 1.42]
Gemcitabine + marimastat	Gemcitabine	0.99 [0.70 - 1.40]
Gemcitabine + aflibercept	Gemcitabine	1.17 [0.84 - 1.61]
Gemcitabine + sorafenib	Gemcitabine	1.27 [0.79 - 2.02]
Gemcitabine + tipifarnib	Gemcitabine	0.97 [0.73 - 1.30]
Gemcitabine + rigosertib	Gemcitabine	1.24 [0.80 - 1.91]
PEFG	Gemcitabine	0.63 [0.32 - 1.25]
Gemcitabine + masitinib	Gemcitabine	0.89 [0.64 - 1.23]
Gemcitabine + elpamotide	Gemcitabine	0.87 [0.47 - 1.62]
Gemcitabine + pemetrexed	Gemcitabine	0.98 [0.74 - 1.30]
Gemcitabine + bevacizumab	Gemcitabine	0.96 [0.72 - 1.27]

© 2022 Takumoto Y et al. *JAMA Network Open*.

B) Hazard rations for progression free survival

Comparison arm	Control arm	PFS-HR (95%CL)
FOLFIRINOX	Gemcitabine	0.47 [0.33 - 0.66]
S-1	Gemcitabine	1.09 [0.80 - 1.49]
Gemcitabine + S-1	Gemcitabine	0.65 [0.52 - 0.82]
Gemcitabine + nab-paclitaxel	Gemcitabine	0.69 [0.51 - 0.93]
Gemcitabine + erlotinib	Gemcitabine	0.77 [0.57 - 1.04]
Gemcitabine + bevacizumab + erlotinib	Gemcitabine	0.56 [0.36 - 0.87]
Gemcitabine + capecitabine	Gemcitabine	0.80 [0.66 - 0.95]
Gemcitabine + cisplatin	Gemcitabine	0.97 [0.71 - 1.32]
Gemcitabine + oxaliplatin	Gemcitabine	0.78 [0.55 - 1.09]
Gemcitabine + cetuximab	Gemcitabine	0.93 [0.70 - 1.24]
Gemcitabine + axitinib	Gemcitabine	1.01 [0.71 - 1.43]
Gemcitabine + marimastat	Gemcitabine	0.95 [0.67 - 1.36]
Gemcitabine + aflibercept	Gemcitabine	1.02 [0.74 - 1.40]
Gemcitabine + sorafenib	Gemcitabine	1.04 [0.65 - 1.65]
Gemcitabine + tipifarnib	Gemcitabine	0.97 [0.72 - 1.30]
Gemcitabine + rigosertib	Gemcitabine	0.96 [0.63 - 1.46]
PEFG	Gemcitabine	0.51 [0.31 - 0.84]

© 2022 Takumoto Y et al. *JAMA Network Open*.

eTable 4: AIC / BIC in estimated GEM curve

Regimen	Model	OS		PFS	
		AIC	BIC	AIC	BIC
Gemcitabine	Exponential	6685.9	6701.0	5668.3	5683.5
	Weibull	6695.0	6710.2	5503.1	5523.3
	Gamma	6858.8	6868.9	5501.6	5516.8
	Lognormal	6735.8	6751.0	5506.4	5521.6
	Gompertz	6699.6	6714.8	5762.3	5777.5
	Loglogistic	6674.6	6694.8	5763.3	5773.4
	Gengamma	6830.4	6845.6	5720.2	5735.4

OS: Overall survival, PFS: Progression free survival, AIC: Akaike's Information Criterion, BIC: Bayesian information criterion

eFigure 1: Network diagram for network meta-analysis

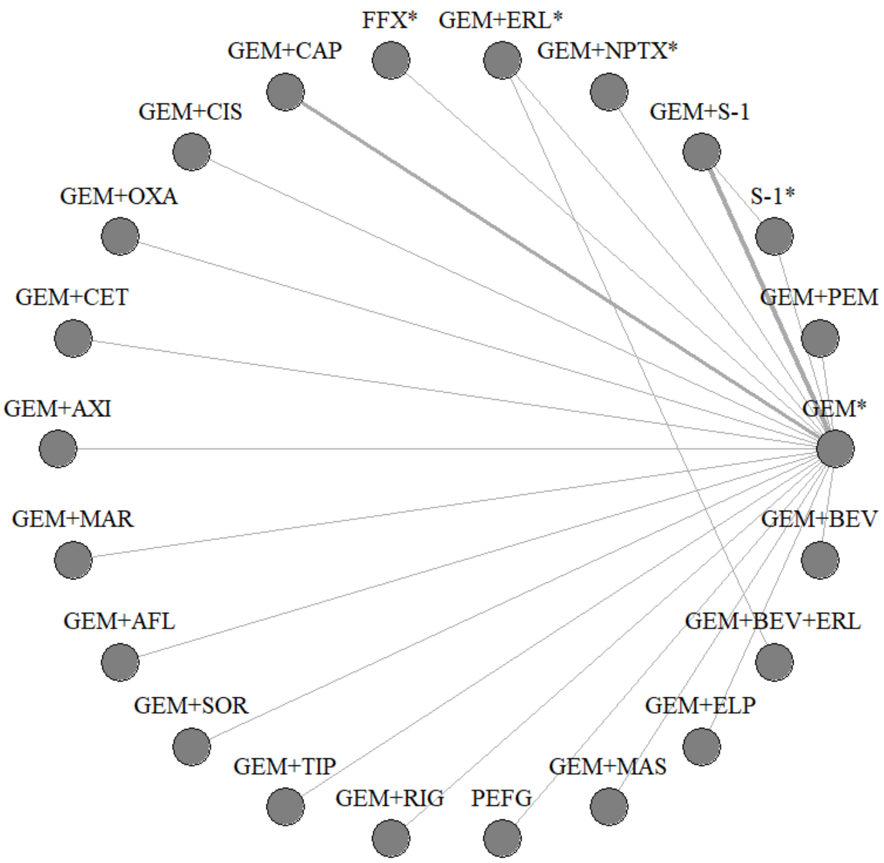

AFL: aflibercept, AXI: axitinib, BEV: bevacizumab, CAP: capecitabine, CET: cetuximab, CIS: cisplatin, ELP: elpamotide, ERLO: erlotinib, FFX: FOLFIRINOX, GEM: gemcitabine, MAS: masitinib, NPTX: nab-paclitaxel, OXA: oxaliplatin, PEM: pemetrexed, PEFG: cisplatin+epirubicin+5-FU+gemcitabine, SOR: sorafenib, TIP: tipifarnib

© 2022 Takumoto Y et al. *JAMA Network Open*.

eFigure 2: HR Ranking of each regimen in NMA

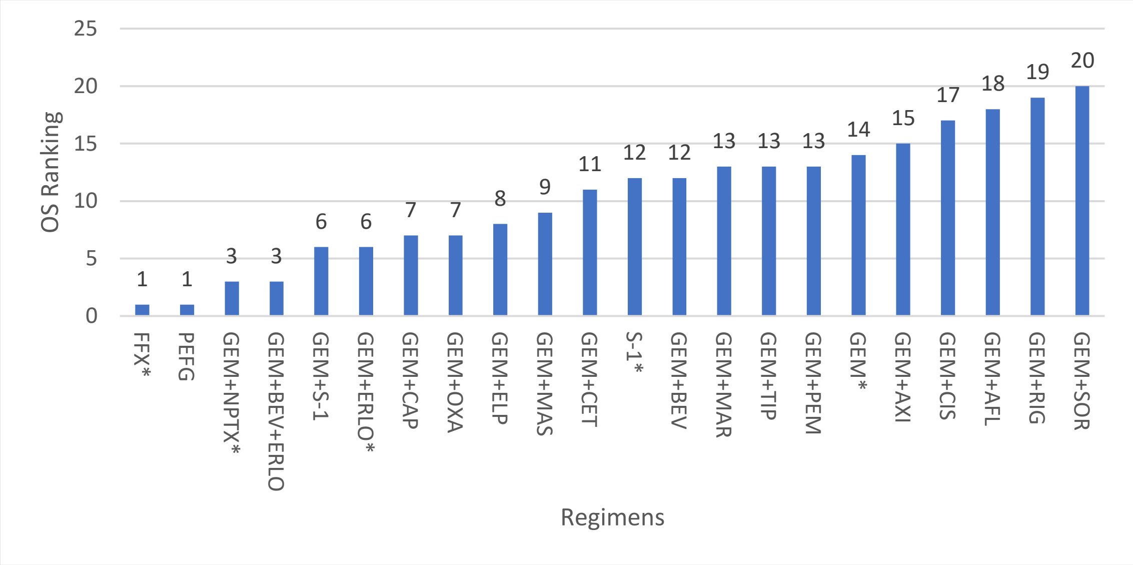

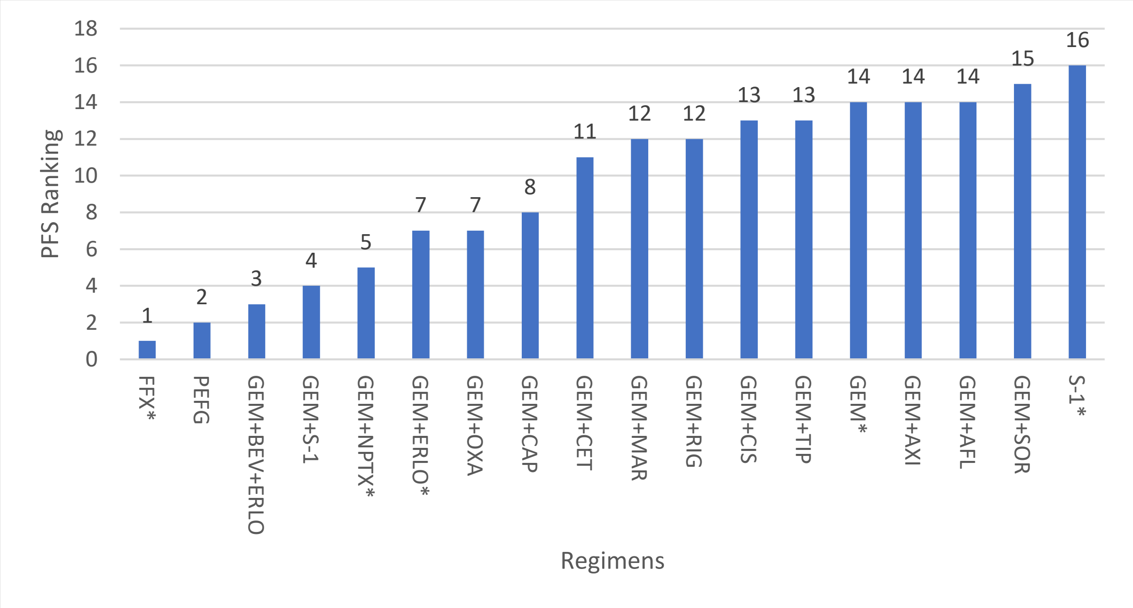

OS: Overall survival, PFS: Progression free survival, AFL: aflibercept, AXI: axitinib, BEV: bevacizumab, CAP: capecitabine, CET: cetuximab, CIS: cisplatin, ELP: elpamotide, ERLO: erlotinib, FFX: FOLFIRINOX, GEM: gemcitabine, MAS: masitinib, NPTX: nab-paclitaxel, OXA: oxaliplatin, PEM: pemetrexed, PEFG: cisplatin+epirubicin+5-FU+gemcitabine, SOR: sorafenib, TIP: tipifarnib

© 2022 Takumoto Y et al. *JAMA Network Open*.

eFigure 3: Model estimation of the KM curves for OS and PFS

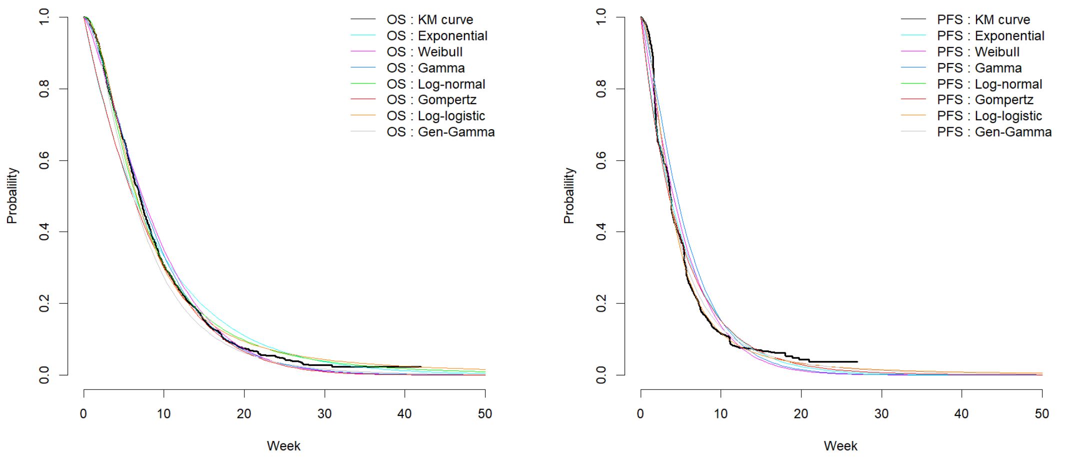

OS: Overall survival, PFS: Progression free survival

© 2022 Takumoto Y et al. *JAMA Network Open*.

eReferences

1. Conroy T, Desseigne F, Ychou M, et al; Groupe Tumeurs Digestives of Unicancer; PRODIGE Intergroup. FOLFIRINOX versus gemcitabine for metastatic pancreatic cancer. N Engl J Med. 2011;364(19):1817-1825.

2. Von Hoff DD, Ervin T, Arena FP, et al. Increased survival in pancreatic cancer with nab-paclitaxel plus gemcitabine. N Engl J Med. 2013;369(18):1691-1703.

3. Ueno H, Ioka T, Ikeda M, et al. Randomized phase III study of gemcitabine plus S-1, S-1 alone, or gemcitabine alone in patients with locally advanced and metastatic pancreatic cancer in Japan and Taiwan: GEST study. J Clin Oncol. 2013;31(13):1640-1648.

4. Nakai Y, Isayama H, Sasaki T, et al. A multicentre randomised phase II trial of gemcitabine alone vs gemcitabine and S-1 combination therapy in advanced pancreatic cancer: GEMSAP study. Br J Cancer. 2012;106(12):1934-1939.

5. Sudo K, Ishihara T, Hirata N, et al. Randomized controlled study of gemcitabine plus S-1 combination chemotherapy versus gemcitabine for unresectable pancreatic cancer. Cancer Chemother Pharmacol. 2014;73(2):389-396.

6. Moore MJ, Goldstein D, Hamm J, et al; National Cancer Institute of Canada Clinical Trials Group. Erlotinib plus gemcitabine compared with gemcitabine alone in patients with advanced pancreatic cancer: a phase III trial of the National Cancer Institute of Canada Clinical Trials Group. J Clin Oncol. 2007;25(15):1960-1966.

7. Louvet C, Labianca R, Hammel P, et al; GERCOR; GISCAD. Gemcitabine in combination with oxaliplatin compared with gemcitabine alone in locally advanced or metastatic pancreatic cancer: results of a GERCOR and GISCAD phase III trial. J Clin Oncol. 2005;23(15):3509-3516.

8. Kindler HL, Niedzwiecki D, Hollis D, et al. Gemcitabine plus bevacizumab compared with gemcitabine plus placebo in patients with advanced pancreatic cancer: phase III trial of the Cancer and Leukemia Group B (CALGB 80303). J Clin Oncol. 2010;28(22):3617-3622.

9. Herrmann R, Bodoky G, Ruhstaller T, et al; Swiss Group for Clinical Cancer Research; Central European Cooperative Oncology Group. Gemcitabine plus capecitabine compared with gemcitabine alone in advanced pancreatic cancer: a randomized, multicenter, phase III trial of the Swiss Group for Clinical Cancer Research and the Central European Cooperative Oncology Group. J Clin Oncol. 2007;25(16):2212-2217.

10. Reni M, Cordio S, Milandri C, et al. Gemcitabine versus cisplatin, epirubicin, fluorouracil, and gemcitabine in advanced pancreatic cancer: a randomised controlled multicentre phase III trial. Lancet Oncol. 2005;6(6):369-376.

11. Cunningham D, Chau I, Stocken DD, et al. Phase III randomized comparison of gemcitabine versus gemcitabine plus capecitabine in patients with advanced pancreatic cancer. J Clin Oncol. 2009;27(33):5513-5518.

© 2022 Takumoto Y et al. *JAMA Network Open*.

12. Philip PA, Benedetti J, Corless CL, et al. Phase III study comparing gemcitabine plus cetuximab versus gemcitabine in patients with advanced pancreatic adenocarcinoma: Southwest Oncology Group-directed intergroup trial S0205. J Clin Oncol. 2010;28(22):3605-3610.
13. Van Cutsem E, Vervenne WL, Bennouna J, et al. Phase III trial of bevacizumab in combination with gemcitabine and erlotinib in patients with metastatic pancreatic cancer. J Clin Oncol. 2009;27(13):2231-2237.
14. Van Cutsem E, van de Velde H, Karasek P, et al. Phase III trial of gemcitabine plus tipifarnib compared with gemcitabine plus placebo in advanced pancreatic cancer. J Clin Oncol. 2004;22(8):1430-1438.
15. Rougier P, Riess H, Manges R, et al. Randomised, placebo-controlled, double-blind, parallel-group phase III study evaluating aflibercept in patients receiving first-line treatment with gemcitabine for metastatic pancreatic cancer. Eur J Cancer. 2013;49(12):2633-2642.
16. Colucci G, Labianca R, Di Costanzo F, et al; Gruppo Oncologico Italia Meridionale (GOIM); Gruppo Italiano per lo Studio dei Carcinomi dell'Apparato Digerente (GISCAD); Gruppo Oncologico Italiano di Ricerca Clinica (GOIRC). Randomized phase III trial of gemcitabine plus cisplatin compared with single-agent gemcitabine as first-line treatment of patients with advanced pancreatic cancer: the GIP-1 study. J Clin Oncol. 2010;28(10):1645-1651.
17. Lee HS, Chung MJ, Park JY, et al; Korean Society of Gastrointestinal Cancer. A randomized, multicenter, phase III study of gemcitabine combined with capecitabine versus gemcitabine alone as first-line chemotherapy for advanced pancreatic cancer in South Korea. Medicine (Baltimore). 2017;96(1):e5702.
18. O'Neil BH, Scott AJ, Ma WW, et al. A phase II/III randomized study to compare the efficacy and safety of rigosertib plus gemcitabine versus gemcitabine alone in patients with previously untreated metastatic pancreatic cancer. Ann Oncol. 2015;26(9):1923-1929. :
19. Deplanque G, Demarchi M, Hebbar M, et al. A randomized, placebo-controlled phase III trial of masitinib plus gemcitabine in the treatment of advanced pancreatic cancer. Ann Oncol. 2015;26(6):1194-1200.
20. Yamaue H, Tsunoda T, Tani M, et al. Randomized phase II/III clinical trial of elpamotide for patients with advanced pancreatic cancer: PEGASUS-PC Study. Cancer Sci. 2015;106(7):883-890.
21. Ozaka M, Matsumura Y, Ishii H, et al. Randomized phase II study of gemcitabine and S-1 combination versus gemcitabine alone in the treatment of unresectable advanced pancreatic cancer (Japan Clinical Cancer Research Organization PC-01 study). Cancer Chemother Pharmacol. 2012;69(5):1197-1204.
22. Bramhall SR, Rosemurgy A, Brown PD, Bowry C, Buckels JA; Marimastat Pancreatic Cancer Study Group. Marimastat as first-line therapy for patients with unresectable pancreatic cancer: a randomized trial. J Clin Oncol. 2001;19(15):3447-3455.
23. Oettle H, Richards D, Ramanathan RK, et al. A phase III trial of pemetrexed plus gemcitabine versus gemcitabine in patients with unresectable or metastatic pancreatic cancer. Ann Oncol. 2005;16(10):1639-1645.

© 2022 Takumoto Y et al. *JAMA Network Open*.

24. Kindler HL, Ioka T, Richel DJ, et al. Axitinib plus gemcitabine versus placebo plus gemcitabine in patients with advanced pancreatic adenocarcinoma: a double-blind randomised phase 3 study. Lancet Oncol. 2011;12(3):256-262.

25. Gonçalves A, Gilabert M, François E, et al. BAYPAN study: a double-blind phase III randomized trial comparing gemcitabine plus sorafenib and gemcitabine plus placebo in patients with advanced pancreatic cancer. Ann Oncol. 2012;23(11):2799-2805.

© 2022 Takumoto Y et al. *JAMA Network Open*.
